# HARMONIZING COGNITIVE SCORES ACROSS WAVES IN THE CHINA HEALTH AND RETIREMENT LONGITUDINAL STUDY

**DOI:** 10.1093/geroni/igae098.2080

**Published:** 2024-12-31

**Authors:** Yingyan Wu, Elizabeth Rose Mayeda, Lindsay Kobayashi, Alden Gross, Yuan Zhang

**Affiliations:** University of California Los Angeles, Los Angeles, California, United States; University of California Los Angeles, Los Angeles, California, United States; University of Michigan, Ann Arbor, Michigan, United States; Johns Hopkins Bloomberg School of Public Health, Baltimore, Maryland, United States; Columbia University, New York, New York, United States

## Abstract

The China Health and Retirement Longitudinal Study (CHARLS) administered a word recall test to assess memory function across five study waves from 2011 to 2020. Since 2018, the word recall test has differed from prior waves in the difficulty of word lists used and number of immediate recall trials given, posing challenges for longitudinal analyses of cognitive change. We thus leveraged weighted equipercentile equating for immediate and delayed recall scores to address nonequivalence in the test across study waves. We created a calibration sample that balanced age, gender, and education to ensure the same underlying test ability across waves. Within the calibration sample, we used equipercentile equating to crosswalk percentile ranks between the scores in the 2015 and 2018 waves and 2015 and 2020 waves, then applied the crosswalk to the full study sample. In the full study sample, the mean original delayed word recall scores were higher in 2018 (4.3 words) and 2020 (5.1 words) versus 2015 (3.2 words). Within the 2018 and 2020 waves, mean original delayed scores were higher than mean original immediate scores (2018: 4.3 vs 3.2, 2020: 5.1 vs. 3.6 words). After applying equipercentile equating, the mean 2018 and 2020 wave immediate and delayed word recall scores were comparable to previous waves (immediate: 2015: 4.1, 2018: 3.7, 2020: 3.8; delayed: 2015: 3.2, 2018: 2.5, 2020: 2.8 words). Equipercentile equating can be used to generate comparable scores to facilitate longitudinal analysis when cognitive test administration procedures change over time.

